# Historical Sketch of the Discovery of the Composition of Water

**Published:** 1879-12

**Authors:** E. G. Betty


### HISTORICAL SKETCH OF THE DISCOVERY OF THE COMPOSITION OF WATER.

BY E. G. BETTY, D. D. S.

At this late day, when the events of near a hundred years ago have taken their place in history, it becomes a matter of no small difficulty to arrive at the truth regarding a disputed honor, when there is naught obtainable but a maze of conflicting accounts, from which one is supposed to extract a clear and just idea of the facts, as they occurred in their proper sequence. As this, in general, is true of most things in the history of a people's development, so also is it true of any one fact, selected almost at random from the great volume of man's recorded deeds. In studying up the history of any particular discovery of importance, there are several things to be taken into consideration before we may hope to attain a true insight into the subject more nearly at hand. We must ascertain first, the general character of a people's mind; the branches or departments of knowledge in which they most excel; the rapidity or tardiness of development in a given direction, and above all, the method of thought pursued in the evolution of scientific knowledge. To do this, the material furnished by the chronicles or annals is to be carefully studied, and such facts as demonstrate a progressive action in the history of mind are to be sifted out, freed from the dross with which they are burthened, and treated not as a confused mass of anecdotes, detailing the schemes and intrigues of courts and kings, but as the pabulum from which a mighty civilization is built.

By classifying and arranging all the evidence thus obtained, the philosophic his'orian is enabled to trace an idea from its inception, and follow it step by step, through all the stages of its growth, gathering strength from each age as it passes along, until at last, with meteoric brilliancy, it bursts upon the world as a grand discovery, marking a new era in the progress of mind, It can be seen then, that any particular discovery to which attention is directed, was foreshadowed by some fact or series of facts discovered previously, the use of which in a comparison of their value, practical and theoretical, giving to the mind a firm basis upon which to extend the scope of its action. The laws marking out the progress of intellectual development are immutable in their nature, and the question of the priority of discovery is a mere matter of time and circumstance in the individual. Each and every one contributes something or other to the common fund of intelligence, and as the pursuit of knowledge is cumulative in its effects upon the developing mind of a people, it is then clearly to be seen that in some one or other individual the facts leading to a great discovery are sure to focus themselves. To determine precisely to whom belongs the credit of having first discovered the composition of water, is a task of such delicacy that even the great Brougham, in his treatment of it, has not given us anything that may be regarded as conclusive. If any one wishes to get a comprehensive idea of the bearings of this controversy, it were better that he made it his study, and from the merits of the case, adduce his own conclusions, for after all, that is the only way in which any satis faction can now be obtained. In view of this state of things, it has been thought proper merely to present such data as are available, without attempting to distort the facts in favor of one contestant or the other, leaving the reader to judge for himself to whom the honor belongs. By the discovery of "fixed air" (carbonic acid gas) in 1752, Black inaugurated a new era in chemistry, and thus directed the attention of his contemporary investigators to the fact that the atmosphere was not the only aeriform body, and that it was possible to find other gaseous substances in nature. The discoveries of hydrogen by Cavendish in 1766; nitrogen by Rutherford in 1772; Oxygen almost simultaneously, by Priestley, of England, and Scheele, of Sweden in 1774, and chlorine by Scheele also in 1774, rapidly followed. A moment's thought will show that this succession of splendid discoveries could not take place without profoundly affecting the scientific world, and fixing the attention of all investigators upon the great strides made in the development of a grand and noble science. For sake of convenience, the experiments relating to the discovery of the composition of water will be given first, followed by a sketch of Mr. Watt's share in the matter. The first observation was made by M. Maquer, a French physician and chemist, in 1776 and 1777, when, during the combustion of "inflammable gas" (hydrogen) he held a porcelain saucer in the flame and found that a moisture adhered to it, which appeared to be water. He also mixed "inflammable gas" and "dephlogisticated air" (oxygen) and described the explosion incident upon their ignition; but from these experiments he seems to have drawn no conclusions. Mr. Warltire in 1781, was the first to fire the mixed gases in a closed vessel by the electric spark, but it was done in the endeavor to determine whether heat was ponderable or not.

Dr. Priestley in the same year performed the same experiment in a glass vessel, and observed the dewy deposit upon the interior, and when Warltire afterwards used glass, he too saw the deposit and said "that it confirmed an opinion he had long entertained, viz: that common air deposits its moisture when phlogisticated" and from this statement, with which it seems Priestley agreed, it appears that both believed it to fully account for the phenomenon. The experiments of Cavendish began in 1781, though after he had heard of Priestley's experience. His investigations were by far the most accurate and scientific, and pursued in a systematic manner. His paper of January, 1784, to the Royal Society, states that his researches were made "with a view to find out the cause of the diminution, which common air is well known to suffer by all the various ways in which it is phlogisticated, and to discover what becomes of the air thus lost or consumed." He mixed hydrogen and common air in a strong glass vessel, and ignited them by the electric spark; all the hydrogen was burned, and a portion of the air, water of course resulted, "also an acid." He had weighed the airs and afterwards the products, and found that the weights corresponded very accurately.

What must have been his conclusion? His biographer says, "thus was effected the important discovery of the composition of water, which Watt had inferred some time before from a careful examination of the similar facts collected by former experimentalists; one of whom, Warltire, had even burned the gases in a close vessel, and by means of electricity. The conclusion arrived at by Mr. Cavendish from his capital experiment was, in his own words, that 'dephlogisticated air is in reality nothing but dephlogisticated water, or water deprived of its phlogiston, or in other words, that water consists of dephlogisticated air united to phlogiston, and that inflammable air is either pure phlogiston, or else water united to phlogiston.' " The combustion of hydrogen and oxygen alone must have more strongly demonstrated the fact to him that the water resulting, could be formed of nothing else than the two gases. The consideration of latent heat in his experiments was altogether neglected, and as will be seen, it bore an important part in the deductions of Watt. The various experiments incident to the discovery of the new gases, and particularly those concerning hydrogen and oxygen, together with Black's doctrine of latent heat, were undoubtedly known to Jas. Watt, and we have now to examine the uses he made of these facts, and point out the method of his inquiry. Being of a philosophic turn of mind, his studies were naturally directed to physics. Having invented the steam engine, or rather made practical the use of steam in driving machinery, he was necessarily familiar with Black's law of latent heat, and made use of it in calculating the available amount of force to be had from the combustion of fuel in his furnaces; the generation of steam and its behavior when subjected to varying degrees of pressure and temperature in the boilers. According to one critic, he had for some years previous to 1783 "been speculating on the subject of water in connection with air, and having, by Black's law of latent heat, associated them together, he was prepared to believe that one is convertible into the other. The idea of an intimate analogy between the two bodies having once entered his mind, gradually ripened, and when at last he completed the discovery, it was merely by reasoning from data which others possessed besides himself." On the tenth of December, 1782, writing to Mr. Boulton, he says: "I have often said that if water could be heated red hot, or something more, it would probably be converted into some kind of air, because steam would in that case have lost all its latent heat, and that it would have been turned solely into sensible heat, and probably a total change of the nature of the fluid would ensue." It appears then, that for many years previous to 1783 Watt entertained the opinion "that air was a modification of water," and on the same date upon which he wrote to Mr. Boulton he is credited with saying that he knew of processes "by which I now believe air is generated from water." The "processes" he mentions undoubtedly had reference to the experiments of Warltire and Priestley; for, be it remembered, these investigations took place early in 1781, and his letter to Gilbert Hamilton, March 26th, 1783, shows that these experiments were known to him, as in fact do some of his letters to Dr. Priestley. It can not be doubted but the experiments being made with oxygen and hydrogen by the several philosophers about this time, drew Watt's attention to them, and his former ideas upon the subject of water and air were brought vividly before his mind, when in these experiments he saw the facts that would establish his theory beyond cavil. Being prepared to believe that water was a compound, he grasped with great readiness the ideas these experiments seemed to indicate, and when sufficient time had elapsed for him to become thoroughly acquainted with the facts as they were developed, his conclusions were rapid and pointed out the truth with remarkable correctness. On the 22d of April, 1783, he again writes to Mr. Hamilton, and after a summary of the facts, beginning with Dr. Priestley's experiments, he deduces the following: "Pure inflammable air is phlogistion itself." "Dephlogisticated air is water deprived of its phlogiston, and united to latent heat." "Water is dephlogisticated air deprived of part of its latent heat, and united to a large dose of phlogiston." To Mr. Fry he gives a recipe for making water, and one of his biographers calls attention to the remarkable fact that the proportions by measure of "pure air and phlogiston" are the same that the chemists of to-day would use, viz. "pure air, one part; phlogiston (hydrogen) two parts." The experiments we have detailed having confirmed his hypothesis, we find in his letter to Dr. Priestley April 26th, 1783, for the first time a definite statement of his theory in its more complete form. After again detailing the recent experiments with the gases, he says: "Let us now consider what obviously happens in the case of the deflagration of the inflammable and dephlogisticated air. These two kinds of air unite with violence; they become red hot aud upon cooling totally disappear. When the vessel is cooled a quantity of water is found in it equal to the weight of the air employed. The water is the only remaining product of the process, and water, light and heat are all the products." "Are we not, then, authorised to conclude that water is composed of dephlogisticated air and phlogiston deprived of part of their latent or elementary heat; that dephlogisticated or pure air is composed of water deprived of its phlogiston, and united to elementary heat and light, and that the latter are contained in it in a latent state, so as not to be sensible to the thermometer or the eye; and if light be only a modification of heat, or a circumstance attending it, or a component part of the .inflammable air, then pure or dephlogisticated air is composed of water deprived of its phlogiston and united to latent heat." This letter Mr. Watt desired to have read before the Royal Society, and for that purpose it was delivered to the president, Sir Joseph Banks. Before this could be done however the author requested a delay, wishing to examine some experiments of Dr. Priestley's. The delay amounted to nearly a year, for the reading did not take place till April 22d, 1784, and it has been the occasion of an acrimonious dispute as to whom really did belong the honor of having discovered the composition of water. This letter was seen and read by many members of the Society during the period between its composition and the final reading, so that there is no doubt at all, but that to Watt belongs the credit of having been the first to draw correct, definite conclusions and reduce them to writing. The discovery of so many gaseous bodies, the large number and variety of experiments that were being made at the time by contemporary philosophers, of course attracted the attention of all men throughout the scientific world and as the importance of their bearing was acknowledged by all, it necessarily became a matter of supreme moment to each contestant that his claims be vindicated. Out of this grew what is known in chemistry as the "Water Controversy."

To establish his own claim to the discovery, Watt was obliged in addition to his paper, to have his letters of April and November, 1783, read before the Royal Society; this was done; the former letter on April 22d, 1784, and the latter on the 29th of the same month. Not taking into account the apparent endeavors of Lavoisier to share the honors of the great discovery, the contest narrows down to two claimants, Cavendish and Watt. That each discovered the fact indedependently and unknown to each other, there can be little doubt; first, because Watt has left evidence conclusive in the shape of letters to show his share in the matter; secondly, from all accounts, Cavendish was a pure and high-minded gentleman and a profound philosopher with a deep regard for the truth for its own sake. When his experiments are studied there can be no doubt that he must have inferred from them the conclusion to which they pointed. Though his paper read before the Royal Society does not contain so precise nor definite a statement as Watt's, it is all the more proof positive that it was solely the product of his own investigations. Besides when we take into consideration the revolution that was going on in the science of chemistry at the time, by reason of the great impulse the developement of mind had acquired since the Reformation, we can not help but know that the discovery was imminent. Cavendish, true to the "empirical character" of the English mind made no hasty conclusions upon insufficient data, nor would he commit himself to anything that he could not demonstrate, hence the absence of any consideration of the part taken by latent heat in the formation of water, because he had some "doubts respecting the nature of heat". Watt on the other hand conceived an idea, speculated "upon the subject of water in connection with air, and having by Black's law of latent heat, associated them together, he was prepared to believe that one is convertible into the other" and when Priestley and Cavendish, especially had made experiments from which the latter could draw no other conclusion. Watt seized upon them and adapted them to a theory, never having made the original experiments himself either to prove or disprove the validity of his hypothesis. His method was analytical, deductive, assuming so and so to be true, and arguing from generalities down to details. Though he made a most felicitous use of that method of study and arrived at truth by it, yet his theory would have fallen to the ground for want of proof had not the facts been demonstrated by some one of an investigating turn of mind. "Both of these inquirers arrived at the truth, but each by a different path." And this antithesis is accurately expressed by one of the most celebrated of living chemists,* who, in his remarks on the composition of water, truly says, that "while Cavendish established the facts, Watt established the idea."

For the facts regarding the discovery, the authorities used are:

Brougham's "Philosophers of the time of George III"; Muirhead's "Life of Jas. Watt"; Buckle's "History of Civilization in England" "Watt's Correspondence on the Composition of Water," as quoted by the above authorities. Liebig's "Letters on Chemistry" as quoted by Buckle.

*Baron von Liebig; since dead.

